# A Systematic Review of the Value Assessment Frameworks Used within Health Technology Assessment of Omics Technologies and Their Actual Adoption from HTA Agencies

**DOI:** 10.3390/ijerph17218001

**Published:** 2020-10-30

**Authors:** Ilda Hoxhaj, Laurenz Govaerts, Steven Simoens, Walter Van Dyck, Isabelle Huys, Iñaki Gutiérrez-Ibarluzea, Stefania Boccia

**Affiliations:** 1 Section of Hygiene, University Department of Life Sciences and Public Health, Università Cattolica del Sacro Cuore, 00168 Roma, Italy; ilda.hoxhaj1@unicatt.it (I.H.); Stefania.boccia@unicatt.it (S.B.); 2 Healthcare Management Centre, Vlerick Business School, 9000 Ghent, Belgium; walter.vandyck@vlerick.com; 3 Department of Pharmaceutical and Pharmacological Sciences, Catholic University of Leuven-KU Leuven, 3000 Leuven, Belgium; steven.simoens@kuleuven.be (S.S.); Isabelle.huys@kuleuven.be (I.H.); 4 Basque Foundation for Health Innovation and Research (BIOEF), Barakaldo, 48902 Basque, Spain; igutierrezibarluzea@bioef.eus; 5 Department of Woman and Child Health and Public Health-Public Health Area, Fondazione Policlinico Universitario A. Gemelli IRCCS, 00168 Roma, Italy

**Keywords:** value assessment frameworks, omics technologies, omics sciences, personalized medicine, health technology assessment, genomics, proteomics, metabolomics, transcriptomics

## Abstract

Background: Omics technologies, enabling the measurements of genes (genomics), mRNA (transcriptomics), proteins (proteomics) and metabolites (metabolomics), are valuable tools for personalized decision-making. We aimed to identify the existing value assessment frameworks used by health technology assessment (HTA) doers for the evaluation of omics technologies through a systematic review. Methods: PubMed, Scopus, Embase and Web of Science databases were searched to retrieve potential eligible articles published until 31 May 2020 in English. Additionally, through a desk research in HTA agencies’ repositories, we retrieved the published reports on the practical use of these frameworks. Results: Twenty-three articles were included in the systematic review. Twenty-two frameworks, which addressed genetic and/or genomic technologies, were described. Most of them derived from the ACCE framework and evaluated the domains of analytical validity, clinical validity and clinical utility. We retrieved forty-five reports, which mainly addressed the commercial transcriptomic prognostics and next generation sequencing, and evaluated clinical effectiveness, economic aspects, and description and technical characteristics. Conclusions: A value assessment framework for the HTA evaluation of omics technologies is not standardized and accepted, yet. Our work reports that the most evaluated domains are analytical validity, clinical validity and clinical utility and economic aspects.

## 1. Introduction

Biological molecules such as DNA, RNA, proteins and metabolites are valuable information sources to inform clinical decision-making [1]. With the advancement of diagnostic technologies, these biological molecules could be exploited in their entirety resulting in the scientific domains: genomics, transcriptomics, proteomics, and metabolomics [2]. These diagnostic technologies or “omics” technologies have become readily available in commercial applications [3]. Two prominent examples in this regard can be considered: Mammaprint and OncotypeDX. These commercial algorithm-based diagnostic tools examine multiple gene transcripts of a tumor in order to assess the prognosis of the patient. They rely on “omics” technologies such as next-generation sequencing and microarray analysis [4,5]. Health technology assessment (HTA) agencies have taken an interest in these technologies, which already offer a significant value to patients and healthcare systems [6]. In particular, applications of “omic” technologies, as the two commercial diagnostic aiding tools specified above, have shown to provide prognostic utility to physicians for better therapy selection in early breast cancer [4,5]. This directly influences patient outcomes and potentially efficient spending in the healthcare system. Commercial diagnostic tests in other indications, including rheumatoid arthritis, diabetes, heart transplantation rejection and other cancerous malignancies, have seen their implementation in the clinic as well. Due to this, the application of these novel “omics” technologies and their added value have been studied and published by different HTA reports [6]. However, as promising as these new technologies are, their assessment seemingly proves to be challenging due to surrounding uncertainty on the clinical utility they provide and their effect on patient outcomes [7]. In addition, from these technologies, a plethora of ethical concerns could arise, which should be considered during the health technology assessment process [7]. To aid the assessment, the use of a value assessment framework for the evaluation can provide value to comprehensively map these issues and identify structural uncertainties to be taken into account.

Value assessment frameworks for diagnostic testing have been developed over the course of almost three decades, mainly within the field of genetic testing, where information from a single gene is acquired for diagnostic, prognostic, or predictive purposes. The advancements of “omics” technologies enable the processing of information on multiple genes, proteins and metabolites, potentially providing valuable information for clinical decision-making. However, as optimistic as the practice may seem, a thorough assessment of these technologies is required before being adopted into the healthcare system, as it should be the case for any technology. The objective of our study was to identify the existing value assessment frameworks used for the evaluation of the omics-based technologies through a systematic review of the literature, and to understand their practical use by HTA agencies.

## 2. Materials and Methods

### 2.1. Systematic Literature Review on the Value Assessment Frameworks

The systematic review was conducted in accordance with the preferred reporting items for systematic reviews and meta-analyses (PRISMA) statement [8]. The protocol of this systematic review was submitted to the International Prospective Register of Systematic Reviews (PROSPERO) and registered as CRD42020168841.

### 2.2. Eligibility Criteria

The search question and the eligibility criteria for inclusion in the systematic review were formulated, addressing the identification of the existing value assessment frameworks used for the evaluation of omics technologies. To address the research questions in a comprehensive and appropriate manner, a commonly used definition of omics sciences, omics technologies, HTA and value assessment frameworks was established. Omics sciences were defined as a set of disciplines related to molecular biology and biochemistry, through which a holistic knowledge can be achieved, in analytical terms, of the features and global content of a biological sample. Omics technologies include genomics, transcriptomics, proteomics and metabolomics. Genomics studies the whole genome of organisms—their function and structure; transcriptomics—the messenger RNA; proteomics—the structure and the functions of the entire proteins; and metabolomics—all chemical processes involving metabolites in a biological cell, tissue, system or organism [2].

Regarding the HTA definition, we used the health technology assessment international (HTAi) referred definition, which considers HTA as “a multidisciplinary process that uses explicit methods to determine the value of a health technology at different points in its lifecycle, aiming to inform decision-making in order to promote an equitable, efficient, and high-quality health system” [9]. Value assessment frameworks referred to systematic thinking of a health technology assessment, aiming the collection, evaluation and organization of the evidence at key research questions [10]. Considering the definitions used for the research question, any article or documents that described a value assessment framework used for the evaluation of omics sciences were considered eligible for inclusion.

### 2.3. Search Strategy

The search strategy was performed in PubMed, Scopus, Web of Science and Embase databases in order to retrieve potential eligible articles published from inception until 31 May 2020.

The search query used in PubMed is:

(Genomic[Title] OR Genomics[Title] OR Proteomic[Title] OR Proteomics[Title] OR Metabolomic[Title] OR Metabolomics[Title] OR Transcriptomic[Title] OR Transcriptomics[Title] OR Omics[Title] OR Omic[Title]) AND (Assay[Title/Abstract] OR assays[Title/Abstract] OR Test[Title/Abstract] OR Testing[Title/Abstract] OR Application[Title/Abstract] OR Applications[Title/Abstract]) AND (Assess*[Title/Abstract] OR Evaluat* [Title/Abstract] OR “Technology Assessment, Biomedical”[Mesh]) AND (Model OR Framework OR Criteria OR Recommendation*).

In the other databases, we adapted the PubMed query according to the search criteria of each database. There were no restrictions applied on study design. We restricted the search to only articles published in the English language, with full-text availability.

### 2.4. Study Selection and Data Extraction

After searching all the databases, the identified articles were uploaded to Mendeley software and duplicates were removed. Two independent researchers (LG; IH) performed the first phase of the screening process, based on titles and abstracts. In the second phase, the selected articles with full text available were carefully read by two researchers (IH; LG) and then, their reference lists were manually screened. Articles that satisfied the eligibility criteria were selected for inclusion. All the steps of the study screening and selection process were reported in a PRISMA flow chart. Any disagreement was resolved by consensus and discrepancies were discussed with a third researcher (IGI).

Two researchers (IH; LG) independently performed the data extraction from each of the included articles. A data extraction form (Table 1) was created to retrieve the following data:

- Author, title, year of publication, the name of the journal/website where the framework is published;
- Institution/organization involved in the development of the framework;
- Type of omics technology evaluated (genomics, proteomics, transcriptomics, metabolomics);
- Name of the framework used and name of the reference framework;
- Evaluation components (format, assessment domain);
- Methodology used for the framework development (literature review, Delphi methods, expert panel, working group consultation).

### 2.5. Desk Research on the HTA Reports

In order to identify the HTA reports on omics technologies published by HTA agencies, we conducted a desk research on the International Network of Agencies for Health Technology Assessment [34] and on the NIHR Center for Reviews and Dissemination [35] websites. The search was restricted only to publicly available reports published in English, Italian, German, Dutch, Portuguese, Spanish and French until May 2020. The desk research on the HTA reports was performed by using the key words “Genomic”, “Genome”, “Genetic”, “Proteomic”, “Metabolomics”, “Transcriptomic”, “Testing”, “Assay”, “ and “Sequencing”.

## 3. Results

### 3.1. Search Results

**Figure 1:**
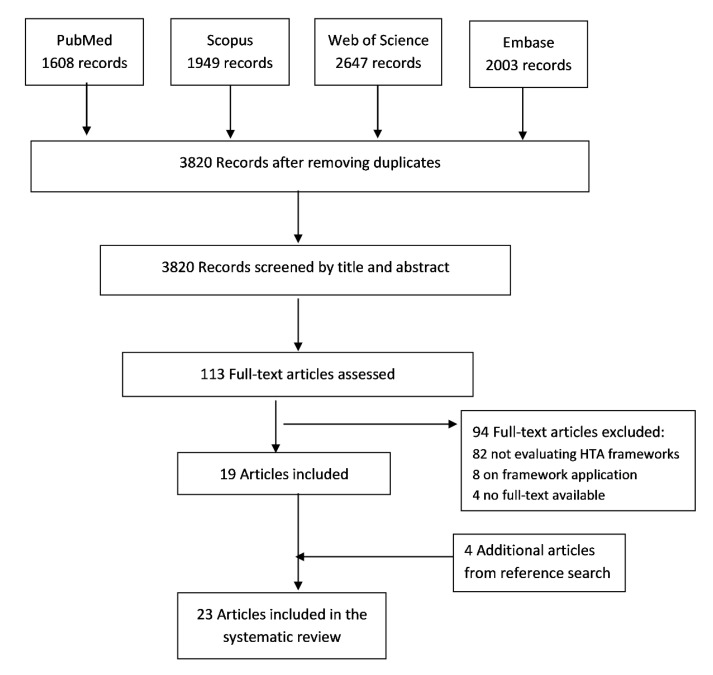
Preferred reporting items for systematic reviews and meta-analyses (PRISMA) flow diagram of the study selection process.

The initial search of PubMed, Scopus, Web of Science and Embase identified a total number of 6946 articles. After removing the duplicates, 3820 articles were screened by title and abstract. One hundred and eight full-text articles were carefully read, of which nineteen articles met our inclusion criteria. Eighty-nine were excluded for not describing a value assessment framework, considering only one evaluation domain, or for not having a full text available. Four articles were included after checking the reference lists of the evaluated full-texts. A total of twenty-three articles [11,12,13,14,15,16,17,18,19,20,21,22,23,24,25,26,27,28,29,30,31,32,33] were included in the systematic review. The entire process of the study screening and selection process in detail is shown in Figure 1.

### 3.2. Characteristics of the Included Studies

The included twenty-three articles described twenty-two value assessment frameworks, which covered the assessment of genetic and/or genomic technologies (Table 1). No frameworks were identified for any other omics technology. The agencies working on these frameworks were based mostly in USA (US Preventive Services Task Force, Centers for Disease Control and Prevention, GAPNET, Cedar Associates LLC, Center for Comparative Effectiveness Research in Cancer Genomics, IFCC Scientific Division Committee on Molecular Diagnostics and Advisory Committee on Heritable Disorders in Newborns and Children), followed by Canada (Agency for Healthcare Research and Quality, McMaster University, Agence d’évaluation des technologies et des modes d’intervention en santé), UK (PHG Foundation, UK Genetic Testing Network), Spain (Junta de Andalucia), Italy (Sapienza University) and EUnetHTA. Ten frameworks addressed genomics testing and genome-based applications, eight frameworks addressed genetic testing, two addressed general personalized medicine technology and one addressed newborn screening testing. Most of the frameworks were derived from the “Analytic validity, Clinical validity, Clinical utility, Ethical, legal and social implications” (ACCE) framework. The main categories of appraisal were clinical utility (14 frameworks), clinical validity (10 frameworks), analytical validity (9 frameworks), and economic evaluation (9 frameworks). Ethical, legal, and societal (ELSI) implications and organizational aspects were considered in six and four frameworks, respectively. The frameworks were developed based on an expert panel consultation (7/22), literature review and expert panel consultation (6/22), or only literature review (4/22), and five articles did not report the methodological process.

### 3.3. Evaluation Frameworks

**Fryback–Thornbury framework**, published in 1991, is considered the first assessment framework. This includes 24 items assessing technical efficiency of a test, diagnostic accuracy efficacy, diagnostic thinking efficacy, therapeutic efficacy, patient outcome efficacy, and societal efficacy. Although this framework has been considered as useful for certain types of screening and diagnostic tests, it has been less useful for assessing genomic diagnostics [27]. The Centre for Disease Control and Prevention (CDC) developed **ACCE framework**, which considers analytical validity, clinical validity, and utility, and ELSI aspects of genetic and genomics testing [12]. The ACCE framework contains a list of 44 key questions and could be considered the first consistently used framework for the assessment of genetic tests. Although it was not the first published framework specifically created for genetic testing, this framework was referenced the most often as the basis for more recently published frameworks (Table 1). A **rapid ACCE model** has been developed with the aim to provide an evaluation in a time frame that meets the decision needs and budgets of stakeholders [30]. This rapid framework, which employs the same 44 key questions used in the original ACCE framework, involves a panel of experts and independent reviewers and is most suitable for topics without a large evidence base [30].

Another framework deriving from the ACCE original framework is the **PHG Foundation framework** [22], created specifically for the UK Genetic Testing Network (UKGTN). This framework amends the ACCE framework in the following ways. Firstly, the PHG framework distinguishes between the assay and the test. The assay is considered the method of determining the presence or quantity of a certain component; therefore, the analytic validity is primarily the matter of the assay. The genetic test relates to the assay but in a clinical situation, hence the context and the purpose of the test is important. The assessment domains of clinical validity and clinical utility thus relates to the genetic test. Secondly, the PHG framework distinguishes two concepts often conflated under the banner of clinical validity—the link between genotype and disease and the evaluation of test performance parameters. Thirdly, the incorporation of different aspects of healthcare quality into the assessment of clinical utility, these include legitimacy, efficacy, effectiveness and appropriateness, as stated by Donabedein et al. [36]. The framework was finally incorporated in the **UKGTN gene dossiers** [32,33]. The gene dossier, developed by the UKGTN based on the ACCE model and the Canadian experience of genetic test evaluation, has evolved since its introduction in 2014 and provides a standardized structure for the presentation of the essential information for genetic tests [32,33].

The ACCE framework was eventually updated by the **Evaluation of Genomic Applications in Practice and Prevention (EGAPP)** working group [13]. The EGAPP working group, part of the CDC, acknowledge that for genetic and genomic tests, a well-structured, standard of systematic evidence review, such as the 44 questions of the ACCE framework, might not fit well for the assessment of these tests. In addition to this, the working group also acknowledges that both the number and quality of studies included in this domain are insufficient. The EGAPP framework therefore allowed for the construction of a chain of evidence, effectively linking crucial elements of the evaluation together through argumentation from analytical validity to clinical validity to finally clinical utility. The EGAPP framework also incorporated some of the elements of the Fryback–Thornbury framework, such as the impact of test utilization on decision-making. This framework provides specific methodological guidance on hierarchies of evidence and considers the overarching question of the assessment to be whether the genetic or genomic test shows clinical utility. From this main question, remaining key questions are identified per assessment. The answers to these key questions are then used to construct the chain of evidence, which finally answers the question of clinical utility. The framework hence provides a more flexible approach to the assessment of genetic or genomic tests as compared to its predecessor, the ACCE framework. This approach also allows for a quantitative assessment of clinical utility through modelling of those genetic or genomic tests, which lack direct evidence [31]. The **risk-benefit framework proposed by Veenstra et al.** further developed this approach [11]. The novel aspect of this framework relies on the utilization of decision analytical modelling techniques to model the effects of the genetic or genomic test, and the projection of several clinical outcomes, such as quality-adjusted life-years. Another framework, **LDT-SynFRAME**, evaluates novel gene-based laboratory developed tests, combining the most pertinent evaluation elements from other assessment frameworks, mostly from ACCE. This new framework included the following components: a clear introductory context, clinical utility, well-substantiated analytical and clinical validity, economic outcomes, ethical aspects, and transparent presentation [18].

A **priority-setting framework**, derived from EGAPP, aimed to prioritize the assessment of candidate genomic technologies in comparative effectiveness research trials. This new framework identified the following nine original priority-setting criteria: health impacts, clinical benefits, economic aspects, analytical and clinical validity, clinical trial implementation and feasibility, ethics and recruitment, market factors and patient-reported outcomes [19].

The most recent framework, published in 2019, was based on ACCE and the non-genetic test EUnetHTA core model^®^ from **Pitini and colleagues** [21]. Along with the ACCE components, it also considered economic evaluation, organizational aspect and delivery models. The latter was not considered in other frameworks for the assessment practice of genetic and genomic tests. The EUnetHTA HTA core model^®^ is perhaps the most versatile and broadly accepted framework for the evaluation of any technology; however, the model seems not well adapted for the evaluation of prognostic tests [15].

Other original frameworks are discussed above. An original framework called the “**three-domain model**” was developed in Canada to guide technology assessment and decision-making with regards to the emerging predictive genetic testing services [25]. The first domain covers evaluative criteria, such as the purpose of the test, effectiveness, costs, demand and cost-effectiveness. The second induces the acceptable cut-off point for each criteria, and the third addresses the need for guidance for conditional coverage decisions. An innovative HTA framework (**INESSS framework**), developed by Blancquaert et al. for genome-based health technologies, covers the assessment of the clinical utility, analytical and clinical validity, acceptability of the screening and diagnostic strategies and the interactions with healthcare services [26]. Another original framework called **“The Genetic testing Evidence Tracking Tool (GETT)”** includes a list of 72 items that needs to be taken into account when evaluating genetic testing implementation. In addition to the items of the ACCE framework, this tool covers the availability of quality-control programs, laboratory and clinical guidelines, and the quality of the supporting data [24]. The **Advisory Committee on Heritable Disorders in Newborns and Children (ACHDNC)** is an original analytic framework that evaluates the specificity of diagnostic and screening tests, potential harms impact, health benefits and costs of diagnosis and treatment [29].

Among all the identified frameworks, ACCE, EGAPP, Fryback–Thornbury framework, EUnetHTA core model and the USPSTF framework have already been piloted and applied in real evaluation projects, considering the needs of patients, payers, regulators, and professional societies, as key stakeholders [37,38].

### 3.4. HTA Reports

**Figure 2:**
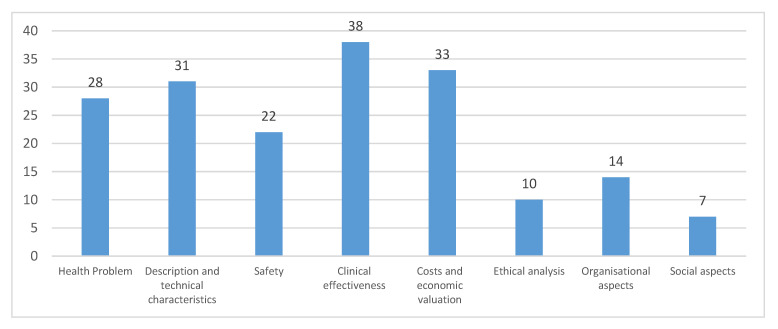
Characteristics of the available forty-five health technology assessment reports on omics technologies.

We identified forty-five HTA reports [39,40,41,42,43,44,45,46,47,48,49,50,51,52,53,54,55,56,57,58,59,60,61,62,63,64,65,66,67,68,69,70,71,72,73,74,75,76,77,78,79,80,81,82,83] conducted from agencies in 15 different countries, mainly in Europe and Canada. The subjects of the assessment were predominantly the commercial transcriptomic prognostics Mammaprint, OncotypeDx, Endopredict and Prosigna (48%), and the genomic technology—next generation sequencing (22%). Based on the definition of HTA, the following evaluation domains in the assessment reports were considered [9]: clinical effectiveness (38/45; 84%); costs and economic evaluation (33/45; 73%); description and technical characteristics (31/45; 69%); health problems (28/45; 62%); safety (49/45; 49%); organizational aspects (14/45; 31%); ethical analysis (10/45; 22%) and social aspects (10/45; 15%). Although most of the HTA reports utilized the concepts within the ACCE evaluation framework, only three reports referenced its use in the methodology (Figure 2). Five reports mentioned EGAPP and only one report mentioned EUnetHTA HTA core model (Supplementary Table S1).

## 4. Discussion

Our systematic review on value assessment frameworks for the evaluation of omics technologies included twenty-three articles that reported twenty-two frameworks on genomics and genetic testing. Most of these frameworks derived from ACCE, focusing on the analytical validity, clinical validity, clinical utility and ELSI aspects. Over the course of recent years, new frameworks have been proposed considering different aspects of the technology during its implementation process. These additional elements cover the organizational aspect, health context, economic evaluation and delivery models. The desk research in the HTA agencies repositories showed that a limited number of them report the use of a value assessment framework in their practice for the evaluation of omics technologies. The majority of these agencies were focused at the assessment of different domains of EUnetHTA HTA core model and a few ACCE components.

Our results indicate that the application of omics sciences in healthcare poses some particular challenges to the HTA process. These challenges include familial implications from disseminating omic information, the design of clinical trials to show clinical utility and the link between the effectiveness of pharmaceuticals (e.g., precision medicines). Identifying these challenges is necessary but not sufficient for decision-makers assessing whether to introduce new sequencing technologies for genomic-based diagnosis. Therefore, further methodological work, supported by technical developments in decision-analytic modeling, is needed [84].

The growth of omics technologies calls for an evaluation and assessment system that is accessible to HTA professionals, managed-care payers, clinicians, and patients. The identified value assessment frameworks may not meet all the required criteria during the evaluation process of omics technologies.

Although numerous omics technologies continue to emerge, to date, most of them have insufficient evidence of clinical validity and utility for their use in clinical practice. The identified frameworks concerned only genetic and genomic testing, probably because genomics are considered as the predecessor of all other omics technologies and the most explored compared to the rest [1]. As of 2019, five transcriptomics assays and only one proteomic assay have ever been translated into clinical practice, showing that translational outcomes are less advanced in other omics fields compared to genomics. Most of the metabolomics studies focus on generating vast amount of data and developing appropriate methods for metabolite measurements, which without an accurate validation decelerate their translational capability in clinical practice [85]. However, the increased availability of more quantitative data and proper validation methods might soon lead to the translations of proteins and transcripts to clinical settings. Although metabolomics, proteomics and transcriptomics are emerging omic technologies, once validated appropriately, they can be used by healthcare professionals as diagnostic tools and also as a tool to assess therapeutic interventions for a more widespread impact on the general population [86].

Despite being more explored, significant gaps exist in the evidence base of genomics, in terms of efficacy, health outcomes, cost-effectiveness, and health services research [87]. Although several frameworks have been proposed over the years to facilitate decision-making for the introduction of genomics tests, some of them refer to tests in general, where others are too narrowly focused on single-gene tests (e.g., screening) or in a particular clinical context (e.g., newborn screening).

These frameworks provide valuable insight into approaching the evaluation of genomic testing; however, each have their limitations. There are important distinctions regarding analytical validity and clinical validity for tests that contain an array of genes, or genes used in combination with other predictors, including clinical factors or protein-based assays.

A single framework might be too general to apply to different clinical scenarios (e.g., screening, diagnosis, prognosis or predictive testing). Considering this, EGAPP proposed different frameworks for the following clinical settings: screening in asymptomatic populations for genetic susceptibility, genetic screening for acquired disease, diagnostic testing for symptomatic disease, and genetic testing to alter therapeutic approaches (e.g., pharmacogenetics) [13]. Furthermore, several of these frameworks have been applied to real HTA evaluation projects, developing recommendations concerning clinical applications and supporting the scientific evidence. The majority of the proposed frameworks derived from ACCE, which is considered somewhat cumbersome, since it addresses 44 different questions. However, ACCE, compared to the other frameworks, has been widely accepted for the evaluation of genetic testing and is considered the basis for framework development. Although the framework was not accredited for in the HTA assessments on omics technologies, the concepts established by ACCE were commonly used throughout the assessments. From our research, cost-effectiveness and budget impact analysis are considered the main decision-assisting tools to inform health policymakers how to best allocate resources. Although the integration of omics technologies in the healthcare system poses ethical issues regarding privacy, informed consent and data sharing, these aspects were not considered in the retrieved reports as organizational and legal aspects, suggesting that these concerns have a small influence in final HTA-related decision-making. Despite the availability of several tools to address ethical aspects in HTA, the lack of this consideration might be due to the difficulty of their application and the subjectivity in ethical analysis [88,89]. Nevertheless, these aspects can, for example, determine the clinical utility of a test and therefore can influence the effectiveness and cost-effectiveness as they are context dependent. It should be therefore noted that the novel definition of HTA therefore explicitly included these aspects: “These dimensions often include clinical effectiveness, safety, costs and economic implications, ethical, social, cultural and legal issues, organizational and environmental aspects, as well as wider implications for the patient, relatives, caregivers, and the population” [9].

The evaluations by HTA agencies on several omics technologies, of which Mammaprint and OncotypeDx were amongst the most frequently assessed, show a repeated evaluation pattern over the course of a decennia, considering that these tests were assessed by the National Institute for Health and Care Excellence (NICE) in 2013 and later again in 2018 by Belgian Kenniscentrum Gezondheidszorg (KCE), the Dutch Zorginstituut Nederland (ZIN)and the Canadian Agency for Drugs and Technologies in Health (CADTH). This repeated assessment pattern illustrates the troublesome issue concerning these diagnostics, the lack of direct evidence to establish their clinical utility and the difficulty in obtaining these data. The practice of HTA agencies regarding omics technologies mainly consists of clinical effectiveness and cost-effectiveness assessment. These domains are reported as the most commonly evaluated in HTA reports of health technologies [90]. The lack of evidence on clinical utility may cast uncertainty on these assessment domains.

Although clinical trials are undertaken to gather this evidence on these tests, healthcare payers have also launched their own initiatives to start covering the tests under a coverage with evidence development (CED) program, thus the future role of real world data and its analysis to produce real world evidence should be carefully considered [4,5,91,92,93]. Evidence is thus continuously gathered, either through clinical trials or through CED programs, which inform later re-assessments of these technologies in their life cycle. This continuous evaluation along the test life cycle should also be considered in value assessment frameworks. With CED programs and early dialogue and early advice between HTA agencies and manufacturers becoming more prominent, HTA agencies’ role is partially shifting from passive evaluators to co-creators of a technology. Value assessment frameworks should therefore reflect this shifting role and also consider the question of whether they are interacting in the life cycle of the omics technology.

The present work, as with any other systematic review, has the potential of publication bias as we included only articles published in English. In order to minimize this, we conducted a rigorous search in the reference list of the evaluated full texts. We were not able to assess the quality of the included articles; however, we evaluated the methodological approach, which showed that the majority of the frameworks were developed based on a specific methodology, such as literature reviews and panel expert consultations. To our knowledge, this is the first attempt concerning the identification of HTA evaluation frameworks for omics sciences, and addressing the practical use of these frameworks by HTA agencies.

Not all the aspects were addressed explicitly among the identified frameworks, which may generate the necessity to create a generalized comprehensive framework, which may result in some challenges due to the heterogeneity of genetic conditions. The rapid advancement of these technologies indicates the need for a continued and regular update of a framework for assessing molecular classifiers.

## 5. Conclusions

We identified twenty-three value assessment frameworks, mostly in the domain of genetic and genomic testing, which could potentially be used for the assessment of omics technologies. However, hardly any frameworks were used for the assessment in the reports produced by HTA agencies. In these reports, the most assessed criteria were clinical effectiveness and cost-effectiveness. A value assessment framework for the HTA evaluation of omics technologies is not standardized and accepted yet. Our work identified the most evaluated domains as analytical validity, clinical validity, clinical utility and economic aspects. Technical description, organizational characteristics and ELSI aspects are not often considered for omics technologies, despite the importance of them due to the consequences of managing omics information not just for the individual person but also relatives.

## Figures and Tables

**Table 1:** Characteristics of the twenty-two value frameworks for the evaluation of omics technologies, retrieved from the twenty-three articles included in the systematic review.

Framework [Reference]	Country	Organization	Year of Publication	Reference Framework	Type of Omics Technology	Evaluation Components	Objective	Framework Website	Methodology Used	Target Audience
Risk-Benefit Framework [11]	USA	Centers for Disease Control and Prevention	2010	EGGAP	genomic testing	1. Decision-analytic modeling 2. Health-related utility 3. Risk-benefit policy matrix	To provide a risk-benefit framework for assessing the health-related utility of genomic tests	NR	stakeholders consultation	NR
ACCE [12]	USA	Centers for Disease Control and Prevention (CDC)	2011	Original framework	genomic screening and diagnostic testing	-Analytic validity, -Clinical validity, -Clinical utility, -Ethical, legal, and social issues	To develop an assessment process for genomic testing, for evaluating, interpreting and reporting genomic data	https://www.cdc.gov/genomics/gtesting/acce/	Expert panel	Systematic reviewers, researchers and policymakers
EGGAP [13]	USA	National Office of Public Health Genomics at the Centers for Disease Control and Prevention,	2009	ACCE	genomic applications	-Analytical validity (analytic sensitivity, analytic specificity, reliability and assay robustness); -Clinical validity (clinical sensitivity and specificity); -Clinical utility (effectiveness)	Outline hierarchy of data sources and study designs to assist in quality rating and assessing evidence	EGGAP working group	literature review and multidiscipli-nary expert panel	Systematic reviewers and policymakers
Practical framework Stages of translational research [14]	USA	N/A	2012	ACCE	genomic testing	Phase 1. Initial test performance and assay refinement; Phase 2. Test validation and generalizability; Phase 3. Clinical test performance and health impacts; Phase 4. Comparison with existing tests Phase 5. Population impacts	To inform where evidence for a particular diagnostic or prognostic test is missing, and to provide clinical guidance	NR	literature review	Manufacturers, test researchers, systematic reviewers, regulators, and other policymakers, clinicians and patients
HTA core model [15]	EU	International	2009	Not reported	Health technology	-Health problem; -Description and technical characteristics; -Safety; -Clinical effectiveness; -Costs and economic valuation; -Ethical analysis; -Organizational aspects; -Social aspects	To develop and test a generic framework to enable international collaboration for producing and sharing results of health technology assessments	https://eunethta.eu/hta-core-model/	Expert panel	Researchers
Framework for the Evaluation of Measures of Genomic Diagnostics [16]	USA	Not reported	2008	ACCE	genomic diagnostic	12 attributes: 1. Priority for maximizing the health of individuals or populations; 2. Economic significance of relevant disease or condition; 3. Genomic diagnostic and drug combination characteristics; 4. Accuracy of genomic diagnostic; 5. Pertinent sub-groups; 6. Pertinent sub-groups; 7. Reproducibility of diagnostic test; 8. Clinical effectiveness and utility; 9. Presentation of indefinite test results; 10. Regulatory status; 11. Economic analyses 12. Patient outcomes and practicability	To propose a novel framework for evaluating the value of genomic diagnostics	NR	Literature review	Policy makers, clinicians, drug developers
Genome-based Knowledge Management in Cycles model (G-KNOMIC) [17]	USA	GAPPNET	2011	ACCE	genomic technology	4 step cycle: (1) knowledge synthesis: aggregate existing research and evidence using explicit and reproducible methods to identify, appraise, and synthesize relevant studies; (2) knowledge evaluation: seeks to understand and measure accuracy, reliability, validity and utility of genomic based services, (3) knowledge implementation: carrying out plans for providing genomic-based services, (4) utilization: uptake and adoption of new genomic-based services by consumers and providers.	To develop a framework for knowledge management that considers knowledge synthesis, evaluation, implementation and utilization, as interconnected elements, essential for the adoption of genomic technologies into practice	NR	Literature review, expert panel	Stakeholders across the public health sector, including researchers, practitioners, policymakers, and educators
SynFRAME [18]	USA	Cedar Associates LLC	2011	ACCE	gene-based laboratory developed tests (LDTs)	-Analytical validity -Clinical validity (study design, population, clinical meaningfulness, statistical significance); -Clinical utility -Economic and social implications -Presentation	To develop an accessible set of criteria that can provide a roadmap for the appraisal of gene-based laboratory developed tests (LDTs)	NR	Systematic review	Policymakers, test developers, researchers, regulators,
A novel framework for stakeholder-informed prioritization of cancer genomics research [19]	USA	Center for Comparative Effectiveness Research in Cancer Genomics (CANCERGEN)	2012	EGAPP	genomic testing	-Population impact; -Analytical/clinical validity; -Potential clinical benefits; -Economic impacts; -Clinical trial implementation and feasibility; -Market factors -Patient-reported outcomes, -Clinical trial ethics -Trial recruitment	To provide a novel framework for stakeholder-informed prioritization of cancer genomics research	NR	A modified Delphi method with stakeholder advisory group including patients/consumers, payers, clinicians, and test developers	Stakeholders, policymakers, patients, payers, and technology developers.
Completeness framework [20]	USA	Not reported	2019	ACCE	elective genomic testing	Analytical performance and interpretive components	To propose a framework designated completeness that evaluates both analytical and interpretative components of genomic tests.	NR	NR	professional community, consumers, providers, laboratories, patients, and consumers
New framework [21]	Italy	N/A	2019	ACCE	genetic testing	1. Genetic tests: -test and clinical condition overview -clinical condition -analytic validity -clinical validity -clinical utility -personal utility 2. Delivery models -delivery models overview -organizational aspects -economic evaluation -ethical, legal and social implications patient perspective 3. Research priorities: evidence gaps& research priorities 4. Decision points: -net benefit -cost-effectiveness -feasibility	To develop a comprehensive new framework that includes an assessment of service delivery	NR	Systematic review and Delphi	Researchers, HTA agencies,
PHG New framework [22]	UK	PHG Foundation	2007	ACCE	Genetic Tests	1. evaluation of the assay 2. evaluation of clinical validity, including clinical test performance 3. evaluation of clinical utility, including test purpose (legitimacy, efficacy, effectiveness, appropriateness), and the feasibility of test delivery (acceptability, efficiency, optimality, equity)	To propose an approach to the evaluation of the genetic tests that expand on and moves beyond the ACCE framework	https://www.phgfoundation.org/documents/369_1409657043.pdf	Expert panel	Policymakers
Framework for the Assessment of Genetic Testing in the Andalusian Public Health System [23]	Spain	Junta de Andalucia	2006	ACCE	Genetic Tests	1. Analytical Validity 2. Clinical validity 3. Clinical utility 4. Ethical Implications 5. Social repercussions 6. Economic and Organisational Impact	The establishment of frameworks and methods to assess new genetic tests, with the aim of defining the services portfolio within the scope of medical genetics	https://pdfs.semanticscholar.org/8ae8/a5263d9775c3442bba2d9b71a3266ee06711.pdf	NR	Decision-makers
GETT: a Genetic testing Evidence Tracking Tool [24]	USA	International Federation of Clinical Chemistry and Laboratory Medicine (IFCC)–IFCC Scientific Division Committee on Molecular Diagnostics	2010	ACCE	Genetic Tests	1. Disease characteristics 2. Comparators 2. Analytical validity 3. Clinical validity 4. Costs 5. Quality improvement program 6. Clinical utility 7. Impact on Healthcare system/(organizational Aspects) 8. Accessibility 9. Social aspects 10. Ethical Aspects	To develop a detailed checklist on the evidence to be produced to evaluate the potential usefulness of a new molecular diagnostic test	https://doi.org/10.1515/CCLM.2010.291	Systematic review and expert panel	Researchers, clinicians, scientists, policymakers
Ontario advisory committee on coverage decisions for new predictive genetic tests [25]	Canada	McMaster university	2003	original framework	Genetic Tests (Predictive)	DOMAIN I: 1. Purpose of the test 2. Effectiveness 3. Additional effects 4. cost-effectiveness 5. costs 6. Demand DOMAIN II: Acceptable cut-offs for decisions DOMAIN III: Decisions under uncertainty	To develop an evaluation model to guide public coverage of new predictive genetic tests in Ontario	/	NR	Policymaker, decision maker
INESSS Framework [26]	Canada	Agence d’évaluation des technologies et des modes d’intervention en santé	2001	HTA	genome-based health applications	Prevalence penetrance (clinical manifestation) Clinical validity Clinical Utility	NR	https://www.inesss.qc.ca/	NR	NR
Fryback-Thornbury Evaluation Framework [27]	Canada	Agency for Healthcare Research and Quality	1991	original framework	Tests in general “Considers all techno-logies”	Technical Efficacy Diagnostic Accuracy Diagnostic Thinking efficacy Therapeutic efficacy Patient Outcome Efficacy Societal Efficacy	To provide an efficacy, hierarchical model that could be applied to all the diagnostic technologies	https://doi.org/10.1177/0272989X9101100203	NR	Policymakers, clinicians, decision makers
The United States Preventive Services Task Force (USPSTF) Evaluation Model [28]	Canada	US Preventive Services Task Force	2001	original framework	Any technology	1. Benefit 2. Harm 3. LSE	Use of analytic frameworks to specify the linkages and key questions connecting the preventive service with health outcomes.	https://www.uspreventiveservicestaskforce.org/uspstf/	literature review, expert panel	Guideline developers, policymakers
ACHDNC analytic framework [29]	USA	Advisory Committee on Heritable Disorders in Newborns and Children	2010	ACCE	New born screening testing	Health condition Clinical validity Analytic validity Clinical utility Cost effectiveness	To provide a framework with inclusion recommendations for newborn screening	NR	literature review, expert panel	Decision makers
Rapid ACCE [30]	USA	Not reported	2007	ACCE	Gene-based testing	-Analytical validity -Clinical validity -Clinical utility -ELSI	To present the rapid-ACCE model and report our early experience of using the ACCE structure to guide systematic reviews for the rapid evaluation of emerging genetic tests.	NR	Expert panel	Stakeholders, policymakers
EGGAP update [31]	USA	Office of Public Health Genomics (OPHG) of the Centers for Disease Control and Prevention (CDC)	2013	EGGAP	genomic applications	-Analytic validity -Clinical utility -Formal decision analysis	To provide an update on recent revisions to Evaluation of Genomic Applications in Practice and Prevention (EGAPP) methods designed to improve efficiency, and an assessment of the implications of whole genome sequencing for evidence-based recommendation development.	NR	Literature review,	Stakeholders
UKGTN Gene Dossier [32,33]	UK	UK Genetic Testing Network (UKGTN)	2007	ACCE	genetic tests	-laboratory details of the test -test characteristics -clinical details of the condition -prevalence of the condition -purpose of the test -the healthcare context in which the test is to be used -the clinical utility of the test -ethical, legal and social considerations -the cost of the test	To evaluate genetic tests and recommend which tests will be provided by the National Health Service	Not reported	Expert panel	Policymakers; Decision makers Regulators

Abbreviations: NR: Not reported; ACCE: analytic validity, clinical validity, clinical utility, ethical, legal and social implications; EGAPP: evaluation of genomic applications in practice and prevention; N/A: not available; HTA: health technology assessment.

## Supplementary Materials

The following are available online at https://www.mdpi.com/1660-4601/17/21/8001/s1, Table S1: Characteristics of the available forty-five Health Technology Assessment Reports on omics-technologies.

## References

[1] Hasin Y., Seldin M., Lusis A. Multi-Omics Approaches to Disease. http://ihec-epigenomes.org/.

[2] Manzoni C., Kia D.A., Vandrovcova J., Hardy J., Wood N.W., Lewis P.A., Ferrari R. Genome, Transcriptome and Proteome: The Rise of Omics Data and Their Integration in Biomedical Sciences. https://pubmed.ncbi.nlm.nih.gov/27881428/.

[3] Karczewski K.J., Snyder M.P. Integrative Omics for Health and Disease. https://pubmed.ncbi.nlm.nih.gov/29479082/.

[4] Cardoso F., van’t Veer L.J., Bogaerts J., Slaets L., Viale G., Delaloge S., Pierga Y.S., Brain E., Causeret S., DeLorenzi M. 70-Gene Signature as an Aid to Treatment Decisions in Early-Stage Breast Cancer. http://www.nejm.org/doi/10.1056/NEJMoa1602253.

[5] Sparano J.A., Gray R.J., Makower D.F., Pritchard K.I., Albain K.S., Hayes D.F., Geyer C.E., Dees E.C., Goetz M.P., Olson J.A. Adjuvant Chemotherapy Guided by a 21-Gene Expression Assay in Breast Cancer. http://www.nejm.org/doi/10.1056/NEJMoa1804710.

[6] Barna A., Cruz-Sanchez T.M., Brigham K.B., Thuong C.T., Kristensen F.B., Durand-Zaleski I. Evidence Required by Health Technolgy Assessment and Reimbursement Bodies Evaluating Diagnostic or Prognostic Algorithms that Include Omics Data. https://pubmed.ncbi.nlm.nih.gov/30136642/.

[7] Love-Koh J., Peel A., Rejon-Parrilla J.C., Ennis K., Lovett R., Manca A., Chalkidou A., Wood H., Taylor M.L. The Future of Precision Medicine: Potential Impacts for Health Technology Assessment. https://pubmed.ncbi.nlm.nih.gov/30003435/.

[8] Liberati A., Altman D.G., Tetzlaff J., Mulrow C., Gøtzsche P.C., Ioannidis J.P.A., Clarke M., Kleijnen J., Devereaux P.J., Moher D. (2009). The PRISMA statement for reporting systematic reviews and meta-analyses of studies that evaluate health care interventions: Explanation and elaboration. J. Clin. Epidemiol..

[9] O’Rourke B., Oortwijn W., Schuller T. (2013). The new definition of health technology assessment: A milestone in international collaboration. Int. J. Technol. Assess Health Care.

[10] Sun F., Bruening W., Erinoff E., Schoelles M.K. Addressing Challenges in Genetic Test Evaluation. Evaluation Frameworks and Assessment of Analytic Validity. www.effectivehealthcare.ahrq.gov/reports/final.cfm..

[11] Veenstra D.L., Roth J.A., Garrison L.P., Ramsey S.D., Burke W. A Formal Risk-Benefit Framework for Genomic Tests: Facilitating the Appropriate Translation of Genomics into Clinical Practice. /pmc/articles/PMC3312796/?report=abstract.

[12] Haddow J.E., Palomaki G.E. An Introduction to Assessing Genomic Screening and Diagnostic Tests. http://www.cdc.gov/genomics/gtesting/ACCE/FBR/.

[13] Teutsch S.M., Bradley L.A., Palomaki G.E., Haddow J.E., Piper M., Calonge N., Dotson W.D., Douglas M.P., Berg A.O., EGAPP Working Group (2009). The evaluation of genomic applications in practice and prevention (EGAPP) initiative: Methods of the EGAPP working group. Genet. Med..

[14] Lin J.S., Thompson M., Goddard K.A.B., Piper M.A., Heneghan C., Whitlock E.P. (2012). Evaluating genomic tests from bench to bedside: A practical framework. BMC Med. Inf. Decis. Mak..

[15] Lampe K., Mäkelä M., Garrido M.V., Anttila H., Autti-Rämö I., Hicks N.J., Hofmann B., Koivisto J., Kunz R., Kärki P. (2009). The HTA Core Model: A novel method for producing and reporting health technology assessments. Int. J. Technol. Assess Health Care.

[16] Issa A.M. (2008). Evaluating the value of genomic diagnostics: Implications for clinical practice and public policy. Adv. Health Econ. Health Serv. Res..

[17] Arar N., Knight S.J., Modell S.M., Issa A.M. (2011). The genome-based knowledge management in Cycles model: A complex adaptive systems framework for implementation of genomic applications. Per. Med..

[18] Hornberger J., Doberne J., Chien R. (2012). Laboratory-developed test-SynFRAME: An approach for assessing laboratory-developed tests synthesized from prior appraisal frameworks. Genet. Test Mol. Biomark..

[19] Esmail L.C., Roth J., Rangarao S., Carlson J.J., Thariani R., Ramsey S.D., Veenstra D.L., Deverka P. (2013). Getting our priorities straight: A novel framework for stakeholder-informed prioritization of cancer genomics research. Genet. Med..

[20] Lu J.T., Ferber M., Hagenkord J., Levin E., South S., Kang H.P., Strong K.A., Bick D.P. (2019). Evaluation for Genetic Disorders in the Absence of a Clinical Indication for Testing: Elective Genomic Testing. J. Mol. Diagn. Elsevier.

[21] Pitini E., D’Andrea E., De Vito C., Rosso A., Unim B., Marzuillo C., Federici A., Di Maria E., Villari P. (2019). A proposal of a new evaluation framework towards implementation of genetic tests. PLoS ONE.

[22] Burke W., Zimmern R. Moving Beyond Acce: An Expanded Framework for Genetic Test Evaluation a Paper for the United Kingdom Genetic Testing Network This Report Is the Result of Work Funded by the Department of Health for the UK Genetic Testing Network (UKGTN). www.phgfoundation.org.

[23] Márquez Calderón S., Briones Pérez de la Blanca E. Framework for the Assessment of Genetic Testing in the Andalusian Public Health System. www.juntadeandalucia..

[24] Rousseau F., Lindsay C., Charland M., Labelle Y., Bergeron J., Blancquaert I., Delage R., Gilfix B., Miron M., Mitchell G.A. (2010). Development and description of GETT: A Genetic testing Evidence Tracking Tool. Clin. Chem. Lab. Med..

[25] Giacomini M., Miller F., Browman G. (2003). Confronting the “gray zones” of technology assessment: Evaluating genetic testing services for public insurance coverage in Canada. Int. J. Technol. Assess Health Care.

[26] Blancquaert I. (2006). Managing partnerships and impact on decision-making: The example of health technology assessment in genetics. Community Genet..

[27] Fryback D.G., Thornbury J.R. (1991). The efficacy of diagnostic imaging. Med. Decis. Mak..

[28] Harris R.P., Helfand M., Woolf S.H., Lohr K.N., Mulrow C.D., Teutsch S.M., Atkins D., Methods Work Group, Third US Preventive Services Task Force (2001). Current methods of the U.S. preventive services task force: A review of the process. Am. J. Prev. Med..

[29] Calonge N., Green N.S., Rinaldo P., Lloyd-Puryear M., Dougherty D., Boyle C., Watson M., Trotter T., Terry S.F., Howell R.R. (2010). Committee Report: Method for Evaluating Conditions Nominated for Population-based Screening of Newborns and Children. Genet. Med..

[30] Gudgeon J.M., McClain M.R., Palomaki G.E., Williams M.S. (2007). Rapid ACCE: Experience with a rapid and structured approach for evaluating gene-based testing. Genetics in Medicine. Nat. Publ. Group.

[31] Veenstra D.L., Piper M., Haddow J.E., Pauker S.G., Klein R., Richards C.S., Tunis S.R., Djulbegovic B., Marrone M., Lin J.S. (2013). Improving the efficiency and relevance of evidence-based recommendations in the era of whole-genome sequencing: An EGAPP methods update. Nat. Publ. Group.

[32] Sanderson S., Zimmern R., Kroese M., Higgins J., Patch C., Emery J. How Can the Evaluation of Genetic Tests be Enhanced? Lessons Learned from the ACCE Framework and Evaluating Genetic Tests in the United Kingdom. https://www.nature.com/articles/gim200597.

[33] Kroese M., Zimmern R.L., Farndon P., Stewart F., Whittaker J. How Can Genetic Tests be Evaluated for Clinical Use? Experience of the UK Genetic Testing Network. www.nature.com/ejhg.

[34] INAHTA International HTA Database–INAHTA. https://www.inahta.org/hta-database/.

[35] NIHR Centre for Reviews and Dissemination NIHR Centre for Reviews and Dissemination–CRD Database. http://www.crd.york.ac.uk/CRDWeb/ResultsPage.asp.

[36] Donabedian A. (1988). The quality of care. How can it be assessed?. JAMA J. Am. Med. Assoc..

[37] Berg A.O., Armstrong K., Botkin J., Calonge N., Haddow J., Hayes M., Kaye C., Phillips K.A., Piper M., Richards C.S. (2009). Recommendations from the EGAPP working group: Can UCT1A1 genotyping reduce morbidity and mortality in patients with metastatic colorectal cancer treated with irinotecan?. Genet. Med..

[38] Berg A.O., Armstrong K., Botkin J., Calonge N., Haddow J., Hayes M., Kaye C., Phillips K.A., Piper M., Richards C.S. (2009). Recommendations from the EGAPP Working Group: Genetic Testing Strategies in Newly Diagnosed Individuals with Colorectal Cancer Aimed at Reducing Morbidity and Mortality from Lynch Syndrome in Relatives. Genet. Med..

[39] García León F.J., Aguado Romeo M.J., Sánchez Jiménez F., Romero Tabares A., Benot López S. Utility of Exome Sequencing for Diagnosed Dismorphic Syndromes, with or without Intellectual Disabilities. https://www.aetsa.org/download/publicaciones/02_AETSA_Exoma_DEF_NIPO.pdf.

[40] Van den Bulcke M., San Miguel L., Salgado R., De Quecker E., De Schutter H., Waeytens A., Van Den Berghe P., Tejpar S., Van Houdt J., Van Laere S. (2015). Next generation sequencing gene panels for targeted therapy in oncology and haemato-oncology. https://kce.fgov.be/sites/default/files/atoms/files/KCE_240_Targeted%20therapy_Scientific%20Report.pdf.

[41] Hanquet G., Vinck I., Thiry N. (2018). The Use of Whole Genome Sequencing in Clinical Practice: Challenges and Organisational Considerations for Belgium.

[42] Newton S., Schubert C., Morona J., Fitzgerald P., Merlin T. Genetic Testing for Hereditary Mutations in the RET Gene. August 2013.

[43] Ward S., Scope A., Rafia R., Pandor A., Harnan S., Evans P., Wyld L. (2013). Gene expression profiling and expanded immunohistochemistry tests to guide the use of adjuvant chemotherapy in breast cancer management: A systematic review and cost-effectiveness analysis. Health Technol. Assess.

[44] Jefferson T., Cerbo M., Chiarolla E., Di Maria E., Favarato M., Gillespie F., Lo Scalzo A., Pinotti G., Turchetti D., Perrini M.R. Agenas—HTA Report—“Next Generation Sequencing (NGS)” Roma, Marzo 2017. http://www.salute.gov.it/imgs/C_17_ReportDispositivi_7_documentoInglese_inglese_itemName_0_documentoENG.pdf.

[45] San Miguel L., Dubois C., Gerkens S., Harrison J., Hulstaert F. (2018). MammaPrint^®^ test for personalised management of adjuvant chemotherapy decisions in early breast cancer. https://kce.fgov.be/sites/default/files/atoms/files/KCE_298_Mammaprint_tests_Report.pdf.

[46] Institut National d’Excellence en Santé et en Services Sociaux (INESSS) (2018). Utilisation d’EndoPredictMC et de ProsignaMC dans les cas de Cancer du Sein Invasif Précoce.

[47] Haute Autorite de Sante (2019). Utilité Clinique des Signatures Génomiques dans le Cancer du Sein de Stade Précoce. www.has-sante.fr.

[48] Young C., Argáez C. (2019). Rapid Genome-wide Testing: A Review of Clinical Utility, Cost-Effectiveness, and Guidelines [Internet].

[49] Férez I.M.M., Márquez-Peláez S., Isabel-Gómez R., Beltrán-Calvo C. (2014). Pruebas Genómicas para el Pronóstico de Pacientes con Cáncer de Mama. MammaPrint^®^ y Oncotype DX^®^ TT—Prognostic Genomic Tests in Early Breast: MammaPrint^®^ and Oncotype DX^®^.

[50] Health Council of The Netherlands (2015). Next Generation Sequencing in Diagnosis. The Hague: Health Council of The Netherlands.

[51] Escalona López S., Callejo Velasco D., Blasco J.A., Guerra M. (2012). Evaluación Económica de las Pruebas Genéticas en el Tratamiento del Cáncer de Mama.

[52] Iqwig G. (2019). Proteomanalyse im Urin zur Erkennung einer Diabetischen Nephropathie bei Patientinnen und Patienten mit Diabetes Mellitus und Arteriellem Hypertonus Impressum.

[53] Tiwana S., Smith A., Leggett L., Mackean G., Lorenzetti D., Clement F. (2013). Use of Oncotype DX for Guiding Adjuvant Chemotherapy Decisions in Early Stage Invasive Breast Cancer Patients in Alberta.

[54] Şenocak G. (2014). Oncotype DX in Women and Men with ER-Positive HER2-Negative Early Stage Breast Cancer Who Are Lymph Node-Positive: A Review of Clinical Effectiveness and Guidelines.

[55] Next Generation DNA Sequencing: A Review of the Cost Effectiveness and Guidelines Canadian Agency for Drugs and Technologies in Health: Ottawa, Canada, 6 February 2014. https://www.ncbi.nlm.nih.gov/books/NBK274072/.

[56] Rouleau G., Boily G. (2015). Séquençage Génétique des Cancers, Validité et Utilité Cliniques des Profils Moléculaires Obtenus à L’aide des Technologies de Séquençage de Nouvelle Génération.

[57] Gosselin C. (2016). Utilisation du Test Oncotype DX MD aux Fins de Décision Thérapeutique dans le Contexte du Traitement du Cancer du Sein Infiltrant.

[58] Meleth S., Reeder-Hayes K., Ashok M., Clark R., Funkhouser W., Wines R., Hill C., Shanahan E., McClure E., Burson K. (2014). Technology Assessment of Molecular Pathology Testing for the Estimation of Prognosis for Common Cancers [Internet].

[59] Isola J., Saijonkari M., Kataja V., Lundin J., Hytönen M., Isojärvi J., Mäkinen E. (2013). Geeniprofilointitestien Merkitys Rintasyövän Hoidon Valinnassa.

[60] Fagerlund B.C., Chudasama K.K. (2019). Prosigna Gene Signature to Assess Expected Benefit from Chemotherapy in Breast Cancer. Assessment of Manufacturer’s Submission.

[61] Ontario Health (Quality) (2020). Gene Expression Profiling Tests for Early-Stage Invasive Breast Cancer: A Health Technology Assessment.

[62] Martínez-Férez I.M., Gómez I., Beltrán-Calvo C. (2015). Second Generation Prognostic Genomic Tests in Early Breast Cancer: EndoPredict^®^ & Prosigna^®^.

[63] CADTH Rapid Response Service (2014). Next Generation DNA Sequencing: A Review of the Cost Effectiveness and Guidelines.

[64] Caballero Villarraso J., Márquez Calderón S., Villegas Portero R. (2007). Aplicaciones Clínicas de las Técnicas Proteómicas Clinical Applications of Proteomic Techniques.

[65] Smartt P. (2010). A Comparison of Gene Expression Profiling Tests for Breast Cancer. http://www.healthsac.net/downloads/publications/HSAC31%20Gene%20Expression%20Profiling%20070710%20FINAL.pdf.

[66] Carballido Fernández M., Llanos Méndez A. (2010). Allomap^TM^ Prueba Genética para el Rechazo de Trasplante Cardíaco Allomap^TM^. Genetic Test for Cardiac Transplant Rejection.

[67] Vignatelli L., Negro A., Giovannini T., Pirini G., Trimaglio F., Ballini L. MammaPrint^®^: Test In Vitro per la Valutazione del Rischio Individuale di Sviluppo di Metastasi in Donne Operate per Tumore alla Mammella. Short Report N.5 Agenzia Sanitaria e Sociale Regionale—Regione Emilia-Romagna. Bologna, Novembre 2011. https://assr.regione.emilia-romagna.it/pubblicazioni/short-report/SR5_MammaPrint_it.

[68] Adelaide Health Technology Assessment (2011). Genetic Testing for Hereditary Mutations in the VHL Gene that Cause von Hippel-Lindau Syndrome MSAC Application no 1153 Assessment Report.

[69] Marchionni L., Wilson R.F., Marinopoulos S.S., Wolff A.C., Parmigiani G., Bass E.B., Goodman S.N. (2007). Impact of gene expression profiling tests on breast cancer outcomes. Evid. Rep. Technol. Assess.

[70] Australian Safety and Efficacy Register of New Interventional Procedures—Surgical (ASERNIP-S) Horizon Scanning Technology Horizon Scanning Report Genetic Screening for Familial Hypercholesterolaemia—Australian and New Zeeland Horizon Scanning Network (ANZHSN) August 2007. https://www1.health.gov.au/internet/horizon/publishing.nsf/Content/211ABF81A69CA39DCA2575AD0080F3DC/$File/HORIZON%20SCANNING%20REPORT%20-%20genetic%20screening%20for%20FH.pdf.

[71] San Miguel L., Vlayen J., De Laet C. (2015). Gene Expression Profiling and Immunohistochemistry Tests for Personalised Management of Adjuvant Chemotherapy Decisions in Early Breast Cancer—A Rapid Assessment.

[72] NIHR Horizon Scanning Centre Prostate Cancer Gene 3 (Progensa PCA3) Assay in the Diagnosis of Prostate Cancer. https://database.inahta.org/article/6617.

[73] Melorose J., Perroy R., Careas S. (2015). Sequenciamento Completo Do Exoma Para Investigação Etiológica De Deficiência Intelectual Inespecífica. Statew. Agric. L Use Baseline.

[74] Swedish Council on Health Technology Assessment (2016). Prenatal Diagnosis through Next Generation Sequencing (NGS). SBU Assessments No 247.

[75] Snowsill T., Coelho H., Huxley N., Jones-Hughes T., Briscoe S., Frayling I.M., Hyde C. (2017). Molecular testing for Lynch syndrome in people with colorectal cancer: Systematic reviews and economic evaluation. Health Technol. Assess.

[76] Martínez-Férez I.M., Viguera-Guerra I., RosarioLozano M.P., Benot-López S., AETSA (2018). Plataformas Genómicas de Carácter Pronóstico—Predictivo en el Cáncer de Mama: Actualización de la Evidencia Revisión Sistemática.

[77] Instituts für Qualität und Wirtschaftlichkeit im Gesundheitswesen (IQWiG) (2016). Abschlussbericht IQWiG-[D14-01] Biomarkerbasierte Tests zur Entscheidung für Oder Gegen Eine Adjuvante Systemische Chemotherapie Beim Primären Mamma-Karzinom.

[78] EunetHTA (2017). Genexpressionstest Mammaprint ^®^.

[79] Baños Álvarez E., Llanos Méndez A., Gómez R.I. (2020). Pruebas Genéticas para Determinación de Riesgo Futuro de Enfermedad Cardiovascular.

[80] Varley-Campbell J., Mújica-Mota R., Coelho H., Ocean N., Barnish M., Packman D., Dodman S., Cooper C., Snowsill T., Kay T. (2019). Three biomarker tests to help diagnose preterm labour: A systematic review and economic evaluation. Health Technol. Assess.

[81] Assessment T. (2020). Genome-wide sequencing for unexplained developmental disabilities or multiple congenital anomalies: A health technology assessment. Ont. Health Technol. Assess Ser..

[82] Wild C., Grössmann N. (2019). FoundationOne^®^CDx: Bestimmung von Genetischen Profilen Solider Tumore. Rapid Assessment Nr. 14. https://eprints.aihta.at/1215/1/Rapid_Assessment_014.pdf.

[83] Health Quality Ontario Pharmacogenomic Testing for Psychotropic Medication Selection: A Systematic Review of the Assurex GeneSight Psychotropic Test.

[84] Payne K., Eden M., Davison N., Bakker E. (2017). Toward Health Technology Assessment of Whole-Genome Sequencing Diagnostic Tests: Challenges and Solutions. Personalized Medicine. Future Med. Ltd..

[85] Pinu F.R., Goldansaz S.A., Jaine J. (2019). Translational metabolomics: Current challenges and future opportunities. Metabolites.

[86] Frantzi M., Bhat A., Latosinska A. (2014). Clinical proteomic biomarkers: Relevant issues on study design & technical considerations in biomarker development. Clin. Transl. Med..

[87] Ioannidis J.P.A., Khoury M.J. (2018). Evidence-based medicine and big genomic data. Human Molecular Genetics. Oxf. Univ. Press.

[88] Saarni S.I., Braunack-Mayer A., Hofmann B., van der Wilt G.J. (2011). Different methods for ethical analysis in health technology assessment: An empirical study. Int. J. Technol. Assess Health Care.

[89] Moattar A.S., Asghari F., Majdzadeh R. (2016). Do ethical considerations influence any in HTA reports? A review of reports. Med. J. Islam Repub. Iran.

[90] Draborg E., Gyrd-Hansen D., Poulsen P.B., Horder M. (2005). International comparison of the definition and the practical application of health technology assessment. Int. J. Technol. Assess Health Care.

[91] MTRC (2018). Innovative Payment Schemes for Medical Technologies and In Vitro Diagnostic Tests in Europe.

[92] Ministère des Solidarités et de la Santé Le Référentiel des Actes Innovants Hors Nomenclature de Biologie et D’anatomopathologie (RIHN)-Ministère des Solidarités et de la Santé. https://solidarites-sante.gouv.fr/systeme-de-sante-et-medico-social/recherche-et-innovation/rihn.

[93] RIZIV Terugbetaling Genexpressieprofilering (GEP) Bij Vroegstadium Borstkanker–RIZIV. https://www.inami.fgov.be/nl/professionals/verzorgingsinstellingen/laboratoria/Paginas/terugbetaling-gep-vroegstadium-borstkanker.aspx.

