# Supplementary material for: A Systematic Review of the Value Assessment Frameworks Used within Health Technology Assessment of Omics Technologies and Their Actual Adoption from HTA Agencies
Source: Int J Environ Res Public Health. 2020 Oct 30;17(21):8001. doi: 10.3390/ijerph17218001 (PMC7663163; doi:10.3390/ijerph17218001)
Supplement: Supplementary file 1 [file ijerph-17-08001-s001.pdf]

Table 1. Characteristics of the available forty-five Health Technology Assessment Reports on omics-technologies.

Report name [Reference]	Year of publication	Agency	Country	Technology Evaluated	Famework Reported	Health Problem	Description and technical characterist-ics	Safety	Clinical effectiv--eness	Costs and economic valuation	Ethical analysis	Organisational aspects	Social aspects
Clinical Applications of Proteomic Techniques [64]	2006	Agencia de Evaluación de Tecnologías Sanitarias de Andalucía	Spain	Proteomics	Not Reported	1	1	0	1	0	0	0	0
Prostate cancer gene 3 (Progensa PCA3) assay in the diagnosis of prostate cancer [72]	2006	National Horizon Scanning Center	UK	The Progensa PCA3	Not reported	0	1	1	1	1	0	0	0
Genetic screening for familial hypercholesterolaemia [70]	2007	Australian Safety and Efficacy Register of New Interventional Procedures-Surgical	Australia	Genetic screening	Not Reported	1	1	1	1	1	1	0	0
Impact of gene expression profiling tests on breast cancer outcomes [69]	2008	Agency for Healthcare Research and Quality	USA	Oncotype DX; MammaPrint; Breast Cancer Profiling test	EGAPP	1	1	1	1	1	0	0	0
A comparison of Gene Expression Profiling tests for Breast cancer [65]	2009	Health Services Assessment Collaboration	New Zealand	OncotypeDx, Mammaprint	Not Reported	1	1	0	1	1	0	0	0
MammaPrint®: in vitro test for the evaluation of individual risk of metastasis in surgically treated women for breast cancer [67]	2011	Agencia sanitaria e sociale regionale-Regione Emilia-Romagna	Italy	Mammaprint	Not Reported	0	1	1	1	1	1	1	1

Economic assessment of genetic tests in mamacarcinoma cancer treatment [51]	2011	Health Technology Assessment Unit of Madrid	Spain	OncotypeDx, Mammaprint	Not Reported	0	0	0	1	1	0	0	0
Genetic testing for hereditary mutations in the VHL gene that cause von Hippel-Lindau syndrome [68]	2012	Adelaide Health Technology Assessment	Australia	Genetic testing	Diagnostic assessment framework	1	1	1	1	1	1	0	0
Allomap™. Genetic test for cardiac transplant rejection. [66]	2012	Agencia de Evaluación de Tecnologías Sanitarias de Andalucía	Spain	Allomap™	Not Reported	1	0	1	1	1	0	0	0
Gene expression profiling and expanded immuno-histochemistry tests to guide the use of adjuvant chemo-therapy in breast cancer management: a systematic review and cost-effectiveness analysis [43]	2013	National Institue for Health and Care Excellence (University of Sheffield)	UK	OncotypeDx, Mammaprint, Endopredict, Prosigna	EGAPP	1	0	0	1	1	0	0	0
Importance of gene profiling tests in the choice of treatment for breast cancer [59]	2013	finCCHTA	Finland	OncotypeDx, Mammaprint, Endopredict	ACCE	1	1	0	1	1	0	1	0
Genetic testing for hereditary mutations in the RET gene [42]	2013	Adelaide Health Technology Assessment	Australia	Genetic testing	Diagnostic assessment framework	1	1	1	1	1	1	1	0
Use of OncotypeDX for guiding adjuvant chemotherapy decisions in early stage invasive breast cancer patients in Alberta [53]	2013	Health Technology Assessment Unit of University of Alberta	Canada	OncotypeDx	Not Reported	1	1	1	1	1	0	0	1

Prognostic genomic tests in early breast: MammaPrint® and Oncotype DX® [49]	2014	Agencia de Evaluación de Tecnologías Sanitarias de Andalucía	Spain	MammaPrint; Oncotype DX	Not Reported	1	1	0	1	1	0	0	0
Technology Assessment of Molecular Pathology Testing for the Estimation of Prognosis for Common Cancers [58]	2014	Agency for Healthcare Research and Quality	USA	Molecular Pathology Testing	Not Reported	1	1	1	1	1	1	1	1
Gene expression profiling of 21 genes in breast cancer to quantify the risk of disease recurrence and predict adjuvant chemotherapy benefit [47]	2014	Medical Services Advisory Committee	Australia	OncotypeDx	Not Reported	1	0	1	1	1	0	1	0
Oncotype Dx in Women and Men with ER-Positive Her2 Negative Early Stage Breast Cancer Who are Lymph Node-Positive: A review of Clinical Effectiveness and Guidelines[54]	2014	Canadian Agency for Drugs and Technologies in Health	Canada	OncotypeDx	Not Reported	0	0	0	1	0	0	0	0
Next Generation DNA Sequencing, A review of the cost-effectivenes and Guidelines [63]	2014	Canadian Agency for Drugs and Technologies in Health	Canada	Next Generation Sequencing	Not Reported	0	0	0	1	1	0	0	0
Second generation prognostic genomic tests in early breast cancer: EndoPredict® & Prosigna™ [62]	2015	Agencia de Evaluación de Tecnologías Sanitarias de Andalucía	Spain	EndoPredict®; Prosigna™	Not Reported	1	1	0	1	0	0	0	0

Proteomanalyse im Urin zur Erkennung einer diabetischen Nephropathie bei Patientinnen und Patienten mit Diabetes mellitus und arteriellem Hypertonus [52]	2015	Institut für Qualität und Wirtschaftlich-keit im Gesundheitsw-esen	Germany	Proteomics	Not Reported	1	1	0	1	0	0	0	0
Next Generation Sequencing Gene panels for Targeted Therapy in Oncology and Haemato-Oncology [40]	2015	Belgian Health Care Knowledge Centre	Belgium	Next Generation Sequencing / In (Hemato)-Oncology	Not reported	0	1	1	1	1	0	1	1
Gene Expression Profiling And Immunohistochemistry Tests For Personalised Managment of Adjuvant Chemotherapy Decisions in Early Breast Cancer [71]	2015	Belgian Health Care Knowledge Centre	Belgium	OncotypeDx, Mammaprint	Not Reported	1	1	1	1	1	0	0	0
Next Generation sequencing in diagnostiek [50]	2015	Health Council of the Netherlands	The Netherlands	Next Generation Sequencing	Not Reported	1	1	0	0	1	1	1	0
Gene sequencing of tumours: clinical validity and utility of molecular profiles obtained by next-generation sequencing technologies [56]	2015	l'Institut national d'excellence en santé et en services sociaux	Canada	Next Generation Sequencing	ACCE	0	1	0	1	0	0	0	0

Using The Oncotype DX ® Assay For Therapeutic Decision Making In The Context Of Invasive Breast Cancer Treatment [57]	2016	l'Institut national d'excellence en santé et en services sociaux	Canada	Oncotype DX	Not Reported	0	1	0	1	1	0	0	0
Biomarker-based tests for the decision for or against adjuvant systemic chemotherapy in primary breast cancer [77]	2016	Institut für Qualität und Wirtschaftlichkeit im Gesundheitsw-esen	Germany	MAAA	Not Reported	1	0	0	1	0	0	0	0
Prenatal Diagnosis through Next Generation Sequencing [74]	2016	Swedish Agency for Health Technology Assessment and Assessment of Social Services	Sweden	Next Generation Sequencing / In prenatal Screening	Not Reported	0	1	0	0	0	1	0	1
HTA Report Next Generation Sequencing (NGS) [44]	2017	Agenas	Italy	Next Generation Sequencing	Not Reported	1	1	1	1	1	1	1	1
Genexpressionstest Mammaprint® [78]	2017	EUnetHTA Collaboration	EU	Mammaprint	EUnetHTA Core model	1	1	1	1	0	0	0	0
Utility of exome sequencing for diagnosed dysmorphic syndromes, with or without intellectual disabilities. Literature review Executive summary [39]	2017	Agencia de Evaluación de Tecnologías Sanitarias de Andalucía	Spain	Exome Sequencing	Not Reported	0	0	0	0	0	1	1	0

Molecular testing for Lynch syndrome in people with colorectal cancer: systematic reviews and economic evaluation [75]	2017	National Horizon Scanning Center	UK	Microsatellite instability testing	EGAPP	1	1	1	0	1	0	0	0
Pharmacogenomic Testing for Psychotropic Medication Selection: A Systematic Review of the Assurex GeneSight Psychotropic Test [83]	2017	Ontario Health Quality	Canada	Pharmaco-genomic testing	Not Reported	1	1	0	1	0	0	0	0
Prognostic genomic tests in early breast cancer: up-date of evidence. Executive summary [76]	2018	Agencia de Evaluación de Tecnologías Sanitarias de Andalucía	Spain	MammaPrint, Oncotype DX, EndoPredict and Prosigna	Not reported	0	0	1	1	0	0	0	0
Use Of Endopredict And Prosigna In Early Invasive Breast Cancer [46]	2018	l'Institut national d'excellence en santé et en services sociaux.	Canada	Endopredict, Prosigna	EGAPP	0	1	0	1	1	0	0	0
Mammaprint Test for personalised management of adjuvant chemotherapy decisions in early breast cancer [45]	2018	Belgian Health Care Knowledge Centre	Belgium	Mammaprint	Not Reported	1	0	1	1	1	0	1	0
The use of whole genome sequencing in clinical practice: Challenges and organisational considerations for Belgium [41]	2018	Belgian Health Care Knowledge Centre	Belgium	Whole Genome Sequencing	Not Reported	0	1	0	0	1	0	1	0

FoundationOne® CDx: genetic profiling of solid tumours [82]	2019	Ludwig Boltzmann Institut	Austria	Next-Generation Sequencing	Not Reported	1	0	0	0	1	0	0	0
Rapid Genome-wide Testing: Clinical Utility and Cost-Effectiveness [48]	2019	Canadian Agency for Drugs and Technologies in Health	Canada	Genome-wide Testing	Not Reported	0	0	0	1	1	0	0	0
Complete sequencing of the Exome for Etiological Investigation of intellectual disability of Indeterminate cause [73]	2019	Conitec	Brasil	Sequenciamento de nova geração – sequenciamento completo do exoma	Not Reported	0	1	0	1	1	0	0	0
Clinical utility of genomic signature in Eary-stage breast cancer [47]	2019	Haute autorité de santé	France	MAAA	Not Reported	0	1	0	1	0	0	0	1
Prosigna Gene Signature to Assess Expected Benfeit from Chemotherapy in Breast Cancer [60]	2019	Norwegian Institute of Public Health	Norway	Prosigna	Not Reported	1	1	1	1	1	0	1	0
Three biomarker tests to help diagnose preterm labour: a systematic review and economic evaluation [80]	2019	National Institute for Health Research	UK	Diagnostic tests PartoSur; Actim Partus; Rapid Fetal Fibronectin 10Q Cassette Kit	Not Reported	1	1	1	1	1	0	0	0
Gene Expression Profiling Tests for Early-Stage Invasive Breast Cancer: A Health Technology Assessment [61]	2020	Canadian Agency for Drugs and Technologies in Health AND Ontaria Health Technology Assessment Series	Canada	OncotypeDx, Mammaprint, Endopredict, Prosigna	Not Reported	1	0	1	1	1	0	1	0

Genetic risk prediction test for cardiovascular disease [79]	2020	Agencia de Evaluación de Tecnologías Sanitarias de Andalucía	Spain	Genetic risk prediction test	Not reported	1	1	1	1	1	1	1	0
Genome-Wide Sequencing for Unexplained Developmental Disabilities or Multiple Congenital Anomalies: A Health Technology Assessment [81]	2020	Ontario Health Quality	Canada	Genome-Wide Sequencing	Not Reported	1	1	1	1	1	0	0	0
Total (45)						28	31	22	38	33	10	14	7

Abbreviations: ACCE: Analytic validity, Clinical validity, Clinical utility, Ethical, legal and social implications; EGAPP: Evaluation of Genomic Applications in Practice and Prevention; MAAA: multianalyte assays with algorithmic analysis;
